# Bioanalytical HPLC Applications of In-Tube Solid Phase Microextraction: A Two-Decade Overview

**DOI:** 10.3390/molecules25092096

**Published:** 2020-04-30

**Authors:** Natalia Manousi, Paraskevas D. Tzanavaras, Constantinos K. Zacharis

**Affiliations:** 1 Laboratory of Analytical Chemistry, School of Chemistry, Faculty of Sciences, Aristotle University of Thessaloniki, GR-54124 Thessaloniki, Greece; nmanousi@chem.auth.gr (N.M.); ptzanava@chem.auth.gr (P.D.T.); 2 Laboratory of Pharmaceutical Analysis, Department of Pharmaceutical Technology, School of Pharmacy, Aristotle University of Thessaloniki, GR-54124 Thessaloniki, Greece

**Keywords:** bioanalysis, in-tube, solid phase microextraction, separation, sample preparation, review

## Abstract

In-tube solid phase microextraction is a cutting-edge sample treatment technique offering significant advantages in terms of miniaturization, green character, automation, and preconcentration prior to analysis. During the past years, there has been a considerable increase in the reported publications, as well as in the research groups focusing their activities on this technique. In the present review article, HPLC bioanalytical applications of in-tube SPME are discussed, covering a wide time frame of twenty years of research reports. Instrumental aspects towards the coupling of in-tube SPME and HPLC are also discussed, and detailed information on materials/coatings and applications in biological samples are provided.

## 1. Introduction

Bioanalysis is the chemical analysis of exogenous (mainly drugs, metabolites, biomarkers) or endogenous (i.e., amino acids) compounds in biological samples [1]. Such analyses and consequently the knowledge of the analyte concentration in biological samples are of great importance for the diagnosis and treatment of diseases, drug discovery and development, therapeutic drug monitoring, bioequivalence studies, clinical and forensic toxicology, doping control, etc. [2,3]. However, biological matrices are quite complicated systems that contain many constituents, such as acids, salts, bases, proteins, peptides etc., while their complexity depends on the type and origin of the matrix (whole blood, serum, plasma, saliva, urine, tissue, hair, breath etc.) [4].

In recent years, remarkable progress has been achieved in the development of highly efficient analytical systems for the determination of drugs in biological samples. Despite these efforts, a sample pretreatment step is still required for the extraction and isolation of the analyte of interest from these matrices prior to the end-point analysis. It is considered to be the most labor-intensive and time-consuming step in the analytical workflow and the main bottleneck in the development of selective and sensitive analytical methods [5,6]. Moreover, sample preparation is considered to be responsible for about 30% of errors [7]. An ideal sample preparation technique should be simple, inexpensive, rapid, and capable to isolate and/or preconcentrate the desired compounds while maintaining the analytical data’s quality [8]. 

Among other sample preparation techniques, solid phase microextraction (SPME) is a state-of-the-art, solvent-free technology introduced by Pawliszyn and his coworkers in 1989 [9,10]. SPME is a green alternative sample cleanup tool to traditional liquid–liquid extraction (LLE) and solid phase extraction (SPE) [11]. Due to its versatility, reliability, low cost, and sampling convenience (i.e., on-site sampling), it has been widely used in combination with separation techniques (e.g., Liquid (LC) and Gas Chromatography (GC), and Capillary Electrophoresis (CE)) in both academic research and routine analyses. Up to now, SPME has been effectively utilized in numerous applications in various scientific fields. As a result of its impact, a search in Scopus revealed almost 2000 publications (e.g., research articles, reviews, book chapters) reporting research/applications using SPME.

Sample preparation techniques, when operated in batch mode (off-line), are laborious, time-consuming, and susceptible to analyte loss, particularly if a large number of samples is required to be processed. For this purpose, the development of automated and on-line coupled systems is a predominant trend in modern analytical chemistry [12]. In recent years, many researchers have focused on the automation of sample pretreatment techniques on both conventional and miniaturized scale [13,14]. A miniaturized system normally needs a smaller amount of sample and organic solvent, while an on-line system reduces the sample preparation steps, improving the method accuracy and precision. Additionally, on-line approaches are desirable when the compound of interest is labile or the amount of sample is limited. 

On-line in-tube microextraction was first assembled by Eisert and Pawliszyn as an automated alternative to fiber SPME [15]. This technique was introduced to overcome some difficulties related to the conventional fiber SPME, including fiber fragility, low sorption capacity, bleeding of thick-film coatings, and reduced efficiency for weakly volatile or thermally labile analytes (for GC analysis). Conventional in-tube SPME schemes utilize a piece of an open tubular fused-silica capillary column with a material (stationary phase) coated on its inner surface. In contrast, in fiber SPME, a sorbent coating on the outer surface of a small-diameter solid rod serves as the extraction medium [9]. In in-tube SPME, a small sample volume migrates towards the stationary phase of the capillary tube. The analytes of interest are extracted and concentrated (via adsorption/absorption phenomena) in the stationary phase and finally eluted using a desorption solvent. One of the advantages of in-tube SPME is the automation ability of this technique, which offers continuous extraction, desorption, and injection of the samples into an analytical column using a typical autosampler. Capillary clogging is considered as the main drawback of this technique and therefore the samples being processed (e.g., biological) must be centrifuged and filtered prior to extraction.

Several review articles have been published in recent years on SPME, highlighting various application areas, developments, and materials/coatings [7,11,16,17,18,19,20,21,22,23,24,25,26,27,28,29]. Recently, a review article has been published as an overview of the most cited review articles on SPME covering theory, applications, automation, and future trends [30]. The present article reviews the developments of the on-line in-tube SPME in combination with liquid chromatography covering a timeframe of the last two decades, focusing on bioanalytical applications.

## 2. Instrumental Configurations

**Figure 1:**
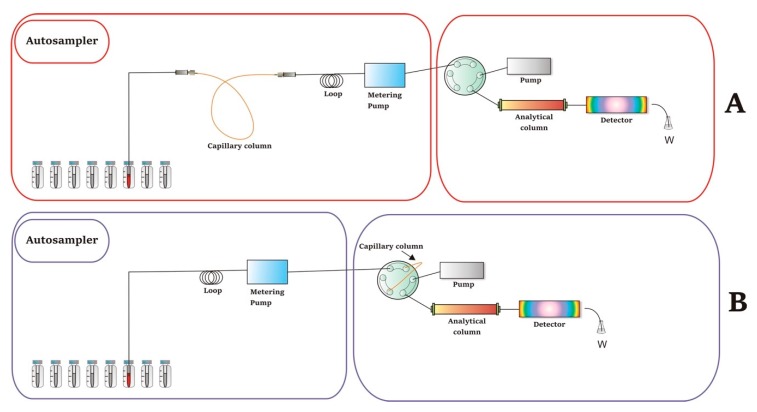
General graphical representation of in-tube SPME setups. (**A**) “Draw-inject” mode; (**B**) “in-valve” mode. It should be noted that the analytical column can be omitted in certain in-tube SPME-MS applications.

Two basic instrumental configurations have been utilized for the implementation of on-line in-tube SPME coupled to HPLC. Figure 1 schematically depicts these setups.

In the first category (Figure 1A), on-line in-tube SPME is performed by placing the capillary column between the needle and the loop of the autosampler. In order to fix the dimensions of the capillary column with the 1/16-inch tubing of the HPLC system, PEEK tubing sleeves are typically utilized and placed at each end of the capillary column. Sample extraction is carried out automatically by performing repeated draw/ejection cycles or in flow-through mode using the autosampler’s software. After the sorption process, the analytes of interest are desorbed and injected into the analytical column using the mobile phase [26]. The extraction efficiency is affected by the sample volume, the number of draw/inject cycles, and the length of the capillary column [31]. When it comes to the analysis of biological samples, the samples must be diluted prior to the SPME process [32].

In the second mode, the capillary extraction tube is positioned to an injection valve via the loop (Figure 1B). The analytes are extracted during the sample loading and transferred to the analytical column by switching the position of the injection valve. Desorption of the analytes can be achieved using either static or dynamic approaches. When a static methodology is followed, the extraction solvent is introduced, and the extracted compounds are transferred to the injection valve of the HPLC system. On the other hand, the dynamic approach involves desorption of the analytes by passing the mobile phase through the extraction column (loop) towards to the analytical column for separation. Although various types of valves can be used in this mode, the six-port, two-position valves are the most abundant [12]. More complicated instrumental setups have been proposed for on-line SPME, including two pumps and/or two six-port valves. Detailed information and features on the operating principles of such configurations have been described recently by M.E.C. Queiroz et al. [23]. 

## 3. Materials and Coatings

**Figure 2:**
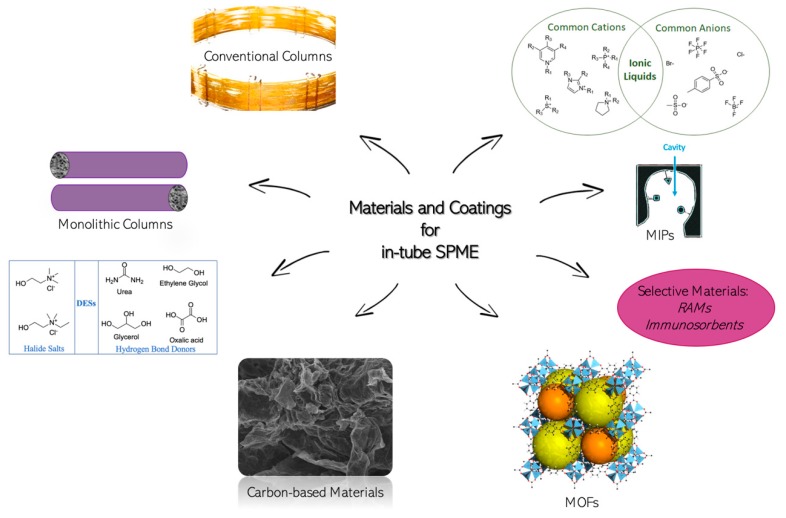
Materials and coatings used in in-tube SPME applications.

Several materials and coatings have been utilized for in-tube SPME bioanalytical applications (Figure 2). In the following sections, the materials that have been utilized for the microextraction of drugs are described.

### 3.1. Conventional Capillary Columns Coating

The main commercial Gas Chromatography (GC) capillary columns that have been used for the in-tube SPME can be divided into wall-coated open tubular (WCOT) and porous layer open tubular (PLOT) columns. The selection of the stationary phase depends both on the properties of the target analyte and on the sample matrix. The selectivity and the sensitivity of the extraction process depend strongly on the coating of the capillary; therefore, it is critical to select the most appropriate extractive phase [27]. Typical examples of WCOT polar columns are 14% cyanopropylphenyl methyl polysiloxane [33,34,35], polyethylene glycol column [34,36,37,38,39,40,41], and 100% nitroterephthalic-modified polyethylene glycol [37]. Typical examples of nonpolar WCOT columns are polydimethyl siloxane [38,39,40,42], 95% polydimethyl siloxane, 5% polyphenyl siloxane [36,38,39,40,43,44,45], 35% diphenyl-65% polydimethylsiloxane [45], 20% diphenyl-80% polydimethylsiloxane [45], and (50%-phenyl)-methylpolysiloxane [46]. Ionic liquid phase commercial SLB-IL100 (Supelco, Bellefonte, PA, USA) has been also evaluated for in-tube SPME [47]. 

Compared to the conventional WCOT liquid-phase GC columns, PLOT columns exhibit a larger adsorption surface and thicker film layer, resulting in higher extraction efficiency of the target analyte. Various PLOT columns have been evaluated, including Carboxen 1006 PLOT capillary column (carbon molecular sieves) (Supelco) [31,32,48,49,50], Rt-U PLOT (divinylbenzene ethylene glycol/dimethylacrylate) (Restek (Bellefonte, PA, USA) [38], CP-Pora PLOT amine (basic modified stylene divinylbenzene polymer) (Varian Inc., Lake Forest, CA, USA) [48], and Supel Q PLOT (Divinylbenzene polymer) (Supelco) [32,36,48,51,52,53,54,55,56].

Inukai et al. reported that although CP-Pora PLOT columns exhibited high extraction efficiency, the stationary phase tended to come off the capillary [48]. This problem was overcome with the use of Carboxen 1006 PLOT column, which also showed great extraction efficiency [31]. Supel Q PLOT columns have been successfully employed for the in-tube SPME of various organic compounds, including heterocyclic amines from hair [36], endocrine disruptors from liquid medicines and intravenous injection solutions [51], melatonin from saliva [52], cortisol from human saliva [53], urinary heterocyclic amines [54], testosterone, cortisol, and dehydroepiandrosterone from saliva [55], and anabolic steroids from urine [56].

### 3.2. Monolithic Capillary Columns

In contrast to conventional packed chromatographic columns, monolithic columns consist of a single piece of porous material that contains macropores and mesopores. Macropores are responsible for the permeability of the monolith, while the presence of mesopores increases the surface area and the loading capacity of the column by providing more active sites. As a result, low back pressure, even at high flow rates, can be obtained [57,58]. Monolithic columns can be divided into silica monoliths, organic polymer monoliths, and organic–inorganic hybrid monoliths [7]. Organic polymer monoliths can be easily prepared from functional and cross-linking monomers, radical initiator and porogenic solvents in order to achieve homogeneous in situ polymerization [27]. Among the benefits of polymer monoliths are the ease in preparation, the good loading capacity, the high surface area, as well as the good porosity control [59].

The most common organic polymer monolithic column is poly(methacrylic acid-ethylene glycol dimethacrylate) (MAA-EGDMA). In this case, methacrylic acid is used as an acidic monomer and ethylene glycol dimethacrylate is the bifunctional crosslinker [60]. MAA-EGDMA monolithic columns have been employed for the extraction of basic drugs from human serum [60], amphetamines from urine samples [61], angiotensin II receptor antagonists from human plasma and urine [62], propranolol enantiomers from human urine [63], telmisartan from rat tissue [64], fluoroquinolones from eggs and albumins [65], amptothecin and 10-hydroxycamptothecin from human plasma [66], sulfonamides from milk [67], ketamine from urine samples [68], quinolones from edible animal food [69], tetracycline antibiotics from fish muscle [70], and amphetamine derivatives from urine [71]. 

Molecularly imprinted polymers, metal–organic frameworks, zeolitic imidazole frameworks, and deep eutectic solvents have been also employed to fabricate monolithic columns for in-tube SPME [72,73,74,75,76,77,78,79]. The application of these materials will be discussed in the respective sections.

Other examples of organic polymer monolithic columns include poly(*N*-vinylcarbazole-co-divinylbenzene) [59], poly(4-vinylpyridine-co-ethylene dimethacrylate) [80], poly(*N*-isopropylacrylamide-co-ethylene dimethacrylate) [81], and poly(vinylphenylboronic acid–co-ethylene glycol dimethacrylate) monolithic material incorporated with graphene oxide [82]. Organic–inorganic hybrid silica monoliths have been also proposed for in-tube SPME. These materials aim to combine the advantages of silica with organic polymer monoliths, resulting in high extraction efficiency [83]. Finally, urea-formaldehyde monolithic columns have been evaluated for hydrophilic in-tube SPME of aminoglycosides [84].

### 3.3. Restricted Access Materials (RAMs)

Restricted-access materials (RAM) fractionate a sample into the protein components and the analyte based on size difference. At the same time, due to size exclusion process, the low-molecular-mass target analyte is extracted and enriched, via partition, into the interior of the stationary phase [27,85,86]. Even though several different structures of RAMs exist, their mechanism of separation is similar. In all cases, a hydrophilic barrier enables the small molecules to permeate through the hydrophobic part of the RAM, while it physically or chemically excludes the macromolecules (e.g., the proteins) [86].

The combination of RAMs and in-tube SPME enables direct and multiple injections of untreated biological samples and the simultaneous protein exclusion and drug preconcentration. However, high column pressure can be obtained in the case of directly highly packed micrometric particles, which can decrease the sample loading speed [27,86]. Moreover, after the extraction step, washing with appropriate solvent is necessary to remove residual proteins from the extraction capillary [85,86].

Mullek et al. used alkyl-diol-silica to prepare a highly biocompatible in-tube SPME capillary for the automated and direct extraction of benzodiazepines from human serum. The porous RAM particles exhibited a hydrophilic electroneutral diol exterior surface that prevents protein adsorption in combination with the size-exclusion mechanism. Due to the bifunctionality of the silica-based RAM, exclusion of matrix proteins took place simultaneously with the preconcentration of benzodiazepines in its hydrophobic porous interior that was modified with C_18_ hydrophobic bonded phase [85]. 

An interesting automated in-tube SPME method has been developed for the determination of interferon alpha 2a in plasma samples using a protein-coated silica RAM sorbent consisting of C_18_ hydrophobic particles [86]. The biocompatible capillary enabled the direct injection of plasma samples, the preconcentration of the target analyte, and the exclusion of coexisting macromolecules. Protein exclusion was mainly attributed to chemical diffusion barrier created by a protein (i.e., bovine serum albumin) network at the outer surface of the particle. The developed RAM capillaries provided good reusability (over 100 times). Compared to the protein precipitation method, the RAM in-tube SPME approach provided a cleaner extract of plasma samples, as well as sufficient preconcentration of the target analyte.

### 3.4. Immunosorbents

Materials that take advantage of antigen–antibody interactions have been employed as sorbents for immunoaffinity in-tube SPME [27]. In this case, specific antibodies are bound to the open fused silica capillary and the extraction of the target analytes is based on the molecular recognition mechanism. The selectivity of immunosorbent depends on the specificity of the immobilized antibodies [87]. Due to the high affinity and selectivity of the antigen–antibody interactions, immunosorbents allow a satisfactory level of selective preconcentration, which is essential for complex biological matrices [88].

There are many approaches for the immobilization of antibodies onto a fused silica surface including covalent binding, noncovalent binding and the sol–gel technique. Covalent binding is the most common approach and it can be easily achieved from the reaction of the free amino groups of the antibody and aldehyde groups on the silica surface after reaction with an aldehyde (e.g., glutaraldehyde) [87].

Antibodies can be divided into monoclonal and polyclonal. Monoclonal antibodies exhibit higher selectivity, especially in the case of large molecules with different binding sites. However, they are generally more expensive than polyclonal antibodies. Compared to the monoclonal antibodies, polyclonal antibodies display higher cross-reactivity [27,87]. The research group of Queiroz used monoclonal antibodies to prepare suitable immunosorbents for the extraction of fluoxetine from serum samples [88], and polyclonal antibodies for the extraction of interferon alpha 2α from plasma samples [87].

In order to increase the phase ratio and the surface area of immunosorbents, Xu et al. synthesized antibody-coated polystyrene nanoparticles immobilized onto the inner capillary wall. The in-tube SPME capillary was employed for the extraction of β2-microglobin and cystatin C. Compared to the monolayer antibody-immobilized capillary, the nanoparticle-coated capillary exhibited almost five times higher extraction capacity [89]. In this work, the immobilization of antibodies onto the surface of the nanoparticles was based on random antibody immobilization (RAI). The RAI technique may lead to a fraction of antibodies with improper orientation toward the surface of the nanoparticles, which can result in a decrease in the potential binding capability. In order to overcome this problem, the same working group designed a poly(glycidyl methacrylate) (PGMA) nanoparticle-coated capillary with oriented antibody immobilization (OAI). Compared to the OAI capillaries without the nanoparticles and the RAI capillaries, the combination of OAI capillaries and PGMA nanoparticles provided higher extraction capacity, as well as lower limits of quantification [90].

### 3.5. Molecularly Imprinted Polymers (MIPs)

Molecularly Imprinted Polymers (MIPs) are synthetic polymeric materials that consist of imprinted sites complementary to a specific molecule. MIPs exhibit high affinity towards analytes with analogous molecular structure, resulting in high extraction selectivity [91]. Molecular recognition is attributed to a combination of size and shape, and hydrogen bonding, electrostatic, and hydrophobic interactions [27]. 

Regarding the preparation of MIPs, the covalent, noncovalent and semicovalent approaches have been reported [92]. In these approaches, the preparation of MIPs is based on the polymerization of a functional monomer and a cross-linker around a template molecule. The template molecule should be able to interact with the functional monomer either covalently or noncovalently in order to develop complexes. A polymerization reaction takes place between the developed complexes and the cross-linker, followed by the removal of the template molecule. As a result, the MIPs contain imprinted sites complementary to the molecular structure and the functional groups of the template molecule. Consequently, MIPs can bind with chemical molecules which are identical or others which are closely related to the template. In order to prevent residual template molecules, extensive washing is required. However, even after many washing steps, the template bleeding problem may still exist. To overcome this drawback, MIPs can be synthesized from templates that are analogues of the target molecules [91]. Compared to the immunosorbents, the synthesis of MIPs requires less time and its cost is lower [92]. Moreover, MIPs are stable at a wider temperature and pH range, they are stable in most organic solvents, and they do not require any special storage conditions [27].

The first in-tube SPME packed capillary column was synthesized from the research group of Pawliszyn by bulk polymerization. For this purpose, propranolol was used as a template molecule and the in-tube capillary was used for the extraction of β-blockers from serum samples [93]. Chaves et al. synthesized a molecularly imprinted sol–gel polymer and evaluated it as stationary phase for the in-tube SPME of interferon alpha 2a from plasma samples. A mild template removal condition using protease was implemented. The developed sorbent exhibited high porosity and large surface area; however, due to the relatively large imprinted cavity, other drugs could also be extracted by the developed MIP sorbent [94]. 

Later, Asiabi et al. synthesized a nanostructured copolymer coating consisting of polypyrrole doped with ethylene glycol dimethacrylate on the inner surface of a stainless-steel tube by electrochemical synthesis. The novel MIP-coated tube was applied for extraction of indomethacin from biological samples [95]. The developed MIP coating was homogeneous, highly cross-linked, and porous with high specific surface, and it was easily prepared.

With the aim to take advantage of the high selectivity of MIPs and the excellent fluid dynamics of the fiber-in-tube, Li et al. [96] designed an online fiber-in-tube SPME technique by packing multiple ofloxacin- and sulfamethazine-imprinted fibers into a PEEK tube. The novel configuration exhibited reduced back pressure, rapid kinetics, and good extraction capacity. Due to the hybrid packing strategy, the developed method was successfully applied for the simultaneous determination of fluoroquinolones and sulfonamides.

Apart from the conventional MIP capillary columns, molecularly imprinted hybrid monolithic capillary columns have been recently developed [72,73,74]. These columns combine the benefits of rigid monolithic columns with the high molecular recognition of MIPs in order to provide highly selective columns with good reproducibility and reusability. MIP monolithic capillary columns have been successfully employed for the in-tube SPME of lysozyme from complex biological samples [72] and 8-hydroxy-2′-deoxyguanosine from urine [73,74]. 

### 3.6. Carbon-Based Materials

Carbon-based nanomaterials such as graphene, graphene oxide, carbon nanotubes, and fullerenes, have recently attracted tremendous interest in analytical chemistry, due to their extraordinary properties [97,98]. As anticipated by their chemical structure, these nanomaterials can interact with hydrogen bonding, π−π stacking, electrostatic forces, van der Waals forces and hydrophobic interactions, resulting in high adsorption efficiency towards the target analytes [99]. 

Carbon fibers were recently used as the sorbent for in-tube SPME. The fibers were placed inside a PEEK tube, providing satisfactory preconcentration, improved peak symmetry, and reduced column dead volume. Moreover, carbon fibers are of low cost, easily accessible, and are characterized by high natural chemical stability [100]. Feng et al. functionalized carbon fibers with graphene oxide (GO) by electrophoretic deposition. Due to the excellent adsorption properties and sufficient surface area of GO, high extraction efficiency was obtained [101].

**Figure 3:**
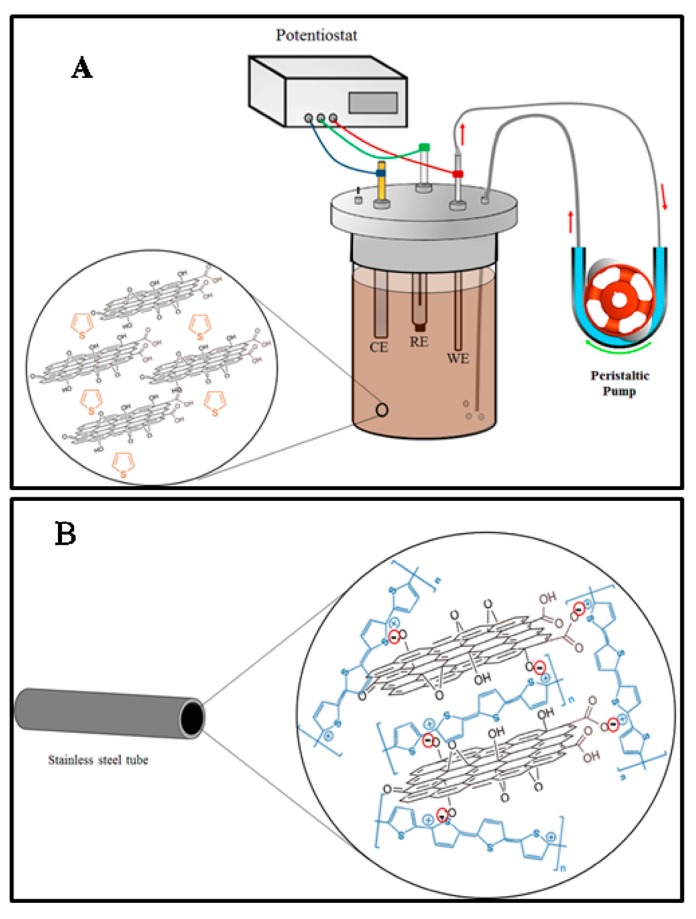
(**A**) Schematic representation of the fabrication process of a polythiophene/graphene (PTh/GO) nanostructured electrodeposited coating using a peristaltic pump. (**B**) Internal surface of stainless steel tube after coating. Adopted from [103] with permissions.

A graphene/polyaniline electrodeposited coating was synthesized and used for the on-line in-tube solid phase microextraction of aldehydes in human exhaled breath condensate. In comparison with the polyaniline coating, the composite material exhibited better mechanical stability, higher specific surface area, good biocompatibility, and long lifespan [102]. Shamsayei evaluated the application of graphene oxide, polythiophene, and graphene oxide/polythiophene composite material as coating for the in-tube SPME of antidepressant drugs. The hybrid coating was prepared by in situ electrodeposition on the inner surface of a stainless-steel tube. Compared to the GO and polythiophene coatings, the composite PTh/GO coating exhibited high specific surface area, as well as long lifetime and good mechanical and chemical stability [103]. An example of the fabrication process of PTh/GO nanostructured electrodeposited coating using a peristaltic pump is illustrated in Figure 3. Other applications of graphene and graphene oxide for in-tube SPME include a graphene oxide–trimethyl-2-methacroyloxyethylammonium chloride-titania (GO-META-TiO_2_) composite monolithic column that was used for the extraction of phosphopeptides [104] and a graphene-embedded porous polymer monolithic column that was used for the extraction of sulfonamides [105]. 

Carbon nanotubes are promising adsorbents for volatile and semivolatile organic compounds due to π-stacking, hydrogen bonding, and hydrophobic interactions. They can be divided into single-walled (SWCNTs) and multiwalled carbon nanotubes (MWCNTs), which consist of one or more sealed tube-shaped layers of graphene, respectively. Adsorption can take place in their easily accessible walls, as well as in their interstitial sites [106]. Several research groups have evaluated the application of SWCNTs and MWCNTs as stationary phases for in-tube SPME. Argente-García et al. evaluated different commercial PDMS-coated capillary columns TRB-35 and TRB-5 (Teknokroma, Barcelona, Spain) with 35% diphenyl-65% polydimethylsiloxane and 5% diphenyl-95% polydimethylsiloxane, respectively. The commercial coatings were used unmodified and functionalized with SWCNTs or MWCNTs, for in-tube SPME of amphetamines as derivatives with 9-fluorenylmethoxycarbonyl chloride (FMOC). It was observed that TRB-35 coating provided higher analytical responses than the TRB-5. Moreover, the introduction of CNTs had a positive effect both on the extraction efficiencies and on the chromatographic profiles. Similar chromatograms were obtained with the SWCNTs and MWCNTs, but MWCNTs provided slightly higher responses [107]. Oxidized multiwalled carbon nanotubes have been also employed for the in-tube SPME of substituted anilines, showing good extraction characteristics [108].

### 3.7. Metal–Organic Frameworks

Metal–organic frameworks (MOFs) are a new class of crystalline porous hybrid organic inorganic supramolecular materials that are based on the coordination of metal ions or clusters with organic linkers. MOFs exhibit a plethora of extraordinary properties, such as high surface area, tunability of pore size and functionality, luminosity, flexibility of their structure, and thermal stability. MOFs have been evaluated for their applications in analytical chemistry, both as sorbents in sample preparation and as stationary phases in high performance liquid chromatography (HPLC), gas chromatography (GC), and capillary electrophoresis (CE) [109,110,111]. Zeolitic imidazolate frameworks (ZIFs) are a subclass of MOFs that combine the benefits of zeolites and MOFs. ZIFs are composed of Zn(II) or Co(II) metal ions and imidazolate and its derivatives as organic linkers [110].

Various MOF and ZIF monolithic columns are promising alternative of polymer and silica monolithic coated capillaries. Those materials combine the benefits of MOFs and monoliths providing efficient coatings with specific surface area, high extraction efficiency and low-backpressure that can be easily prepared [75,76,77,78]. However, since many MOFs exhibit low stability in aqueous solutions, proper selection of the MOF should be performed [110].

Lin et al. evaluated different MOFs including MIL-100(Cr), MIL-101(Cr), MIL-100(Fe), MIL-100(Al), UIO-66(Zr) and MIL-88B(Cr) for the in-tube SPME of penicillins. The researchers synthesized different MOF–polymer columns using ethylene dimethacrylate, butyl methacrylate and an imidazolium-based ionic liquid as porogenic solvent followed by microwave-assisted polymerization with the addition the metal–organic framework. MIL-101(Cr) exhibited the best extraction performance due to its porous structure, intermolecular chromium–π interactions and π–π interactions [75]. Other MOFs that have been employed for in-tube SPME are MIL-53(Al) [76], NH_2_-MIL-53(Al) [78], and ZIF-8 [77].

### 3.8. Ionic Liquids and Deep Eutectic Solvents

Recently, applications of ionic liquids (ILs) and deep eutectic solvents (DESs) have been described. These materials are an alternative to environmentally harmful traditional organic solvents [104,112,113]. ILs are generally composed of bulky, nonsymmetrical organic cations (i.e., imidazolium, pyrrolidinium, pyridinium, ammonium, phosphonium etc.) and different inorganic or organic anions (i.e., tetrafluoroborate anions, bromide anions etc.) [99,114]. DESs are formed from a eutectic mixture of Lewis or Brønsted acids and bases. DESs may contain a variety of anionic and/or cationic species. DESs typically consist of substituted quaternary ammonium salts and hydrogen bond donors. Despite the fact that DESs and ILs show similar physical properties, their chemical properties suggest significantly different applications [115].

ILs were applied to functionalize copper wires and tube for fiber-in-tube SPME of five estrogens. For this purpose, 1-dodecyl-3- vinylimidazolium bromide was applied as the monomer and 1,6-di(3-vinylimidazolium) hexane bibromide was employed as the crosslinking agent to improve the stability of the ILs polymer coating. The crosslinked poly(ionic liquids) coating was grafted onto a copper support through a radical polymerization reaction [116].

A poly(DES-ethylene glycol dimethacrylate) monolithic column based on a green deep eutectic solvent (DES) was prepared for in-tube solid phase microextraction of nonsteroidal anti-inflammatory drugs (NSAIDs) In this case, a choline chloride and itaconic acid DES was adopted as functional monomer to synthesize a polymeric monolith inside polydopamine-functionalized PEEK tube. The DES-based column can interact with the target analyte through hydrophobic interactions, electrostatic interactions, and hydrogen bonding in order to provide high extraction efficiency [79].

### 3.9. Other Materials

Other materials that have been employed as coating materials for capillary columns include β-cyclodextrin [117], titania (TiO_2_) [118], polydopamine/dialdehyde starch/chitosan composite [112], Fe_3_O_4_/SiO_2_/layered double (Cu-Cr) hydroxide nanoparticles [113], Cu-Cr-Al ternary layered double hydroxide/polythiophene coating [119], polypyrrole [120,121,122,123,124,125,126,127,128,129], sol–gel materials [130], and Fe_3_O_4_/SiO_2_ [131].

Polypyrrole (Ppy) and its derivatives have been widely studied as coating materials for capillary columns for in-tube SPME. Polypyrrole can be easily prepared through polymerization from organic or aqueous media through electrochemical or chemical methods [120]. Due to its chemical structure, polypyrrole is expected to extract the target analytes through *π–π*, dipole–dipole, acid–base, hydrogen bonding, ion-exchange, and hydrophobic interactions [121]. Polypyrrole-coated capillaries have been successfully employed for the extraction of verapamil [121], naproxen [122], various drugs for point-of-care (POC) diagnosis [123], stimulants [124], fluoxetine and norfluoxetine enantiomers [125], β-blockers [126], and *N*-nitrosamines [127]. Acidic drugs have been extracted from biological matrices in a nanostructured polypyrrole-dodecyl benzene sulfonate sorbent coated on the inner surface of a stainless steel tube and the surface of the stainless steel particles [120], while acidic, basic, and neutral drugs have been coextracted from biological samples with a copolymer of polypyrrole and indole-2-carboxylic acid (PPy-co-PIca) [128]. Copolymers of polypyrrole exhibit good stability, high surface area, and interaction capability, resulting in high adsorption capacity [128]. A hybrid inorganic–organic zinc oxide/polypyrrole coating material has been also synthesized and evaluated for the online in-tube SPME of monohydroxy polycyclic aromatic hydrocarbons in urine. The hybrid sorbent combined the large surface area of zinc oxide nanorods and the porous structure of polypyrrole to provide a stable coating with a long lifespan [129].

Sol–gel capillary microextraction was initially reported by Mallik et al. in 2002 [132]. The researchers evaluated the application of sol−gel poly(dimethylsiloxane) (PDMS) and sol−gel poly(ethylene glycol) (PEG) for the extraction of nonpolar and polar analytes, respectively. The sol–gel coating was prepared through a single-step procedure and allowed the in situ creation of chemically bonded coatings that are characterized by high thermal and chemical stability. Another sol–gel coating that has been used in bioanalysis is a zirconia-based hybrid organic–inorganic coating [130].

Magnetic in-tube SPME has been also proposed. In this case, a magnetic hybrid material formed by Fe_3_O_4_ nanoparticles supported on SiO_2_ was synthesized and immobilized in the surface of a silica capillary column to obtain a magnetic adsorbent extraction phase. Subsequently, the coated column was placed inside a magnetic coil to enable the application of a magnetic field. Due to the magnetic forces, high extraction efficiency was observed [131].

## 4. Applications

When analyzing biological samples (whole blood, serum, plasma), sample preparation plays an important role, since these samples are quite complex, containing moderate-to-high levels of proteins. Analysis of such matrices generally includes processing of a considerable number of samples, where the concentration of analytes is frequently at quite low levels [133].

Numerous in-tube SPME-based methods have been reported in the literature, analyzing drugs in biological samples (i.e., urine, serum, plasma, saliva, breath and hair). In recent years, there has been a dramatic increase in the pharmaceutical and biomedical applications of this technique, presenting new coating materials and configurations [11,30]. In the following subsections, these applications are summarized, and the characteristics of the approaches employed are discussed. An overview of in-tube SPME applications in bioanalysis during the last 20 years is presented in Table 1. 

### 4.1. Plasma and Serum

Plasma and serum samples typically require deproteinization to prevent clogging of the capillary column. In the majority of the in-tube SPME applications, acetonitrile has been employed to facilitate protein precipitation in plasma samples [33,34,35,80,83,86,103,125,134,135,136,137,138]. Alternatively pure methanol [121,140,141], 1% acetic acid in acetonitrile [79], or mixtures of acetonitrile/methanol 90/10 *v*/*v* have been also utilized for this purpose [139]. Moreover, some approaches followed the direct dilution of plasma samples in 0.1 % *v*/*v* aqueous formic acid solution [37,46], 1 % *v*/*v* aqueous acetic acid solution [40,78,93,144], phosphate buffer [60,66,88,143], or a mixture of phosphate buffer/acetonitrile 90/10 *v*/*v* [62] and phosphate buffer/methanol 95/5 *v*/*v* [85]. An interesting exception against to “protein precipitation” approach is the utilization of RAM materials, which permits the direct injection of biological fluids [85,86]. Such materials enable the simultaneous exclusion of macromolecules (proteins, peptides) by chemical diffusion barrier and drug preconcentration (see Section 3.3).

In many cases, the cleavage of the conjugated forms of the drug and their metabolites from the proteins and fats is mandatory [153]. In such matrices, the drugs are typically at low concentrations, and their stability should be of concern [154]. 

An interesting approach has been proposed by Souza et al. for the determination of endocannabinoids (anandamide, 2-arachidonoyl glycerol) in plasma samples obtained from patients with Parkinson’s disease [136]. The authors used an ionic-liquid-based fused silica capillary column synthesized by thermal-initiated polymerization. The proposed stationary phase showed adequate chemical and mechanical strength, permitting its reuse for more than 90 times without changes in structural integrity, extraction reproducibility, and efficiency. The plasma samples after protein precipitation with CH_3_CN were centrifuged, dried, and reconstituted with a mixture of CH_3_COONH_4_/CH_3_CN prior to SPME protocol. Using a sample volume of 400 μL, the sensitivity of the method was satisfactory for the determination of the analytes in the examined samples. A year later, the same group of authors published a study dedicated to the determination of cannabinoids in plasma using dummy MIP monolithic capillary column as in-tube extraction media [134]. The developed material—after its characterization—was applied to the extraction and quantitation of the analytes in plasma specimens from patients treated with cannabidiol. In order to achieve the best extraction performance, several factors (adsorption, desorption solvents, flow rate, sample volume, washing step, pH value, monolith length) were carefully investigated. Satisfactory linearity in the range of 10–300 ng mL^−1^ was achieved using UHPLC-MS/MS. The analytes were detected in multiple reaction monitoring (MRM) mode, offering high selectivity and sensitivity. 

**Figure 4:**
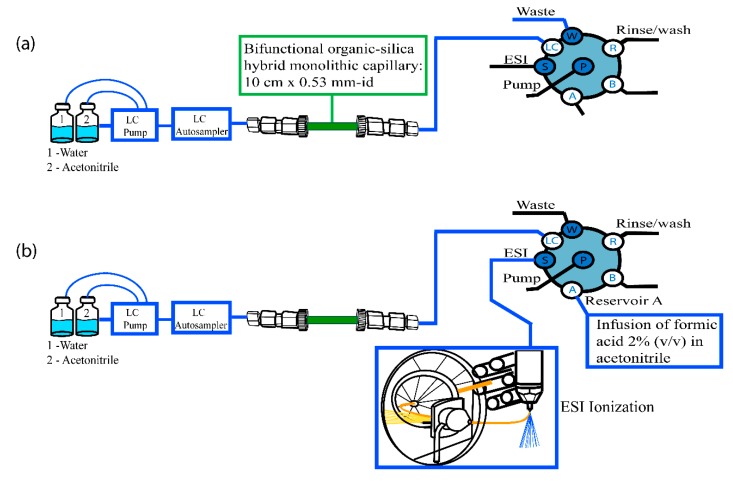
Instrumental configuration of in-tube SPME-MS/MS. (**a**) Sample extraction on monolithic capillary column, (**b**) Elution of the analytes by switching the valve position. Adopted from [137] with permissions.

A monolithic in-tube SPME has been utilized for the analysis of amino acids and neurotransmitters in plasma samples obtained from schizophrenic patients [137]. A bifunctional organic–silica hybrid monolithic capillary having both cyano- and amino-groups enabled the separation of the ionizable analytes. The in-tube SPME column was placed between the autosampler and six-port valve prior to the MS detector (Figure 4). The approach includes three steps: (i) preconcentration of the analyte on the column and simultaneous exclusion of the endogenous compounds using pure acetonitrile; (ii) elution of the analytes using water as mobile phase; and (iii) postcapillary infusion of 2% formic acid in acetonitrile to boost the desolvation capacity and the ionization of the analytes.

Three different liquid chromatographic methods have been published for the determination of interferon alpha 2a in plasma samples using either the “in-valve” [86,94] or “draw-inject” [87] methodology. An HPLC-fluorescence method has been reported by A.R. Chaves et al. [86]. Restricted access material (RAM) has been exploited for the preparation of a biocompatible in-tube SPME capillary. This sorbent permitted the direct injection of biological fluids as well as the simultaneous exclusion of macromolecules (e.g., proteins) by chemical diffusion barrier. The researchers took advantage of using the “draw/inject” methodology to preconcentrate the samples and improve the sensitivity of the method up to 0.06 MIU mL^−1^. For the preparation of the SPME column C_18_ silica particles (45 μm diameter) were used, which are favorable for the isolation of the analyte (*M*_r_ = 19 kDa). According to the authors, capillary clogging was not observed during the entire study, while the RAM columns could be reused more than 100 times without any loss of their efficiency. The method is capable of determining the analyte in the range of 0.06–3 MIU mL^−1^ with satisfactory precision and accuracy. A molecularly imprinted sol–gel polymer (MIP) using protein as template has been fabricated and utilized for in-tube SPME of the analyte [94]. After investigating the parameters affecting the extraction, better efficiency was obtained at a sample volume of 50 μL. A potential disadvantage of the method includes the limited lifetime of the MIP column (only 20 extractions). The method presented linear response over the range of 8–300 ng mL^−1^, which is sufficient for this type of analysis. The authors working on the same topic proposed a different approach from their previous work by preparing an immunosorbent capillary for the isolation of the analyte from plasma samples [87]. Although the sample pH is generally critical, affecting the SPME performance, in this study, it was kept constant in order to maintain sustained antibody–antigen binding and immunoaffinity phase activity. The high affinity and specific recognition of the immunosorbent resulted in a 10-fold lower LOQ compared to their previous report [86].

Two different approaches have been suggested for the in-tube SPME extraction of nonsteroidal anti-inflammatory drugs (NSAIDs), such as ketoprofen, flurbiprofen, and diclofenac [79,80]. The research group of Chen took advantage of using a polymer-based monolithic column synthesized by deep eutectic solvent [79]. The reproducibility of the synthesis of the stationary phase was evaluated by preparing six different batches, where the %RSD of the peak areas of the analytes was less than 4.3%. The method exhibited adequate linearity and satisfactory accuracy for the determination of ketoprofen, flurbiprofen, and diclofenac. One year later, Yu et al. developed an LC/MS method for the quantitation of NSAIDs utilizing a poly(4-vinylpyridine-co-ethylene dimethacrylate)-based monolithic column. During the optimization of the SPME parameters, they concluded that the salting-out phenomenon played a predominant role in the extraction of the analyte. Remarkable preconcentration ability was observed for all compounds, possibly due to the highly hydrophobic character of the SPME sorbent [80]. 

**Figure 5:**
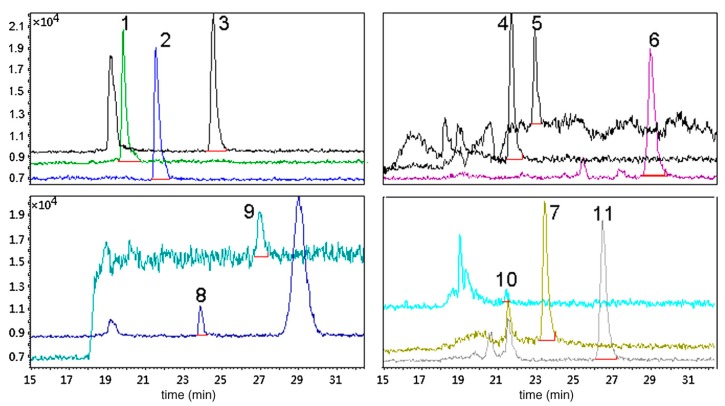
LC/MS SIM chromatograms from the analysis of plasma sample spiked with 10 antidepressant drugs after in-tube SPME (Peaks: 1, trazodone; 2, citalopram; 3, amitriptyline; 4, doxepin; 5, paroxetine; 6, clomipramine; 7, imipramine (internal standard); 8, fluvoxamine; 9, sertraline; 10, clozapine; 11, fluoxetine. Adopted from [83] with permissions.

A group of three tricyclic antidepressants—amitriptyline, imipramine, and chlorpromazine—were determined using electrochemically controlled in-tube SPME [140]. A single compartment equipped with three electrodes was utilized to carry out the electrocopolymerization of a poly(In-Co-Th) material in the inner surface of the SPME capillary. The morphology and the characterization of the material were tested by a series of techniques (SEM, IR, etc.). It was proved that the extraction efficiency remained unaffected after 80 consecutive SPME procedures. The validity of the method has been evaluated in terms of linearity, precision, and accuracy. An analogous electrodeposition approach has been followed by Shamsayei et al. for the synthesis of polythiophene/graphene oxide-based in-tube SPME [103]. Two antidepressant drugs, amitriptyline and doxepin, were determined in human plasma using HPLC-UV. The optimization of the main parameters affecting the extraction performance was carried out using central composite design. The analytical method demonstrated acceptable accuracy (recovery ranged between 91.5% and 97.4%) and precision (% RSD varied between 3.4% and 4.2%). The main disadvantage of the method is the necessity of using a relatively large sample volume (3 mL) to achieve the desired sensitivity. The simultaneous determination of ten antidepressant drugs in plasma samples has been reported using hybrid silica monolith-based in-tube SPME [83]. The cyanoethyl-based SPME column was positioned in a six-port injection valve as a loop prior to the main analytical column of the LC/MS setup. After examining the parameters affecting the separation and the detection of the analytes, successful quantitation was achieved using isocratic elution (0.2% formic acid/CH_3_CN, 70/30% *v*/*v*) on a C_18_ column. In order to minimize the relative recoveries which ranged from 40.5 to 125.3% matrix-matched calibration curves were selected. Some typical LC-MS chromatograms from the analysis of the antidepressants in plasma samples are depicted in Figure 5.

A polypyrrole-coated capillary column has been utilized for the extraction of a racemic mixture of fluoxetine from human plasma samples [125]. The separation of the drug enantiomers has been carried out on a *tris*-(3,5-dimethylphenyl carbamate) cellulose column (Chiralcel OD-R) using a mixture of potassium hexafluorophosphate 7.5 mM and sodium phosphate 0.25 M solution (pH 3.0)/CH_3_CN (75/25, *v*/*v*) as mobile phase. The performance of the synthesized polypyrrole-based sorbent was found to be superior compared to the commercially-available materials (PEG (polyethylene glycol), OV-1701 (14% cyanopropyl phenylmethyl polysiloxane)). The analytes were fluorimetrically detected at λ_ex_/λ_em_ = 230/290 nm. Up to thirty-five samples can be processed within a working day. 

Another recent application of HPLC to the bioanalysis of drugs involves the quantitation of lidocaine and its metabolite (monoethyl glycinexylidide) in plasma samples obtained from pregnant women with gestational diabetes mellitus [33]. The capillary tube was coated with 14% cyanopropylphenyl methylpolysiloxane (OV-1701) and utilized for the extraction of the analytes. Using the “draw/inject” approach, the method is capable of detecting the compounds at a level of 15 ng mL^−1^. The specificity of the method was investigated against seventeen other potential coadministrated drugs. After the necessary protein precipitation step, the extraction and desorption of the analytes were accomplished rapidly within 4 min, while the chromatographic separation was achieved within 15 min. 

An automated on-line in-tube SPME method has been developed for the determination of camptothecin and its natural analogue 10-hydroxycamptothecin [66]. Pure organic solvent as sample diluent could be beneficial for protein precipitation; however, they are not preferable, since they may lead to a decrease in the SPME efficiency. After optimization, methanol content of 4% was selected. The calibration curve of camptothecin was linear in the range of 0.5 to 500 ng mL^−1^. The method demonstrated excellent reproducibility yielding % RSD to less than 1.6% and the LOD (based on S/N = 3) was found to be 0.1 ng mL^−1^. 

A different approach was followed by Ling et al. for the analysis of sulfonamides (sulfadiazine, sulfadimidine, and sulfamethoxazole) in plasma samples by placing electrochemically modified carbon fiber bundles in the PEEK tube [141]. A carbon fiber branch provides large specific surface area and, due to its chemical inertness, can be an ideal substrate for surface modification to get the desired structure. The PEEK tube loop was placed in the injection valve of the HPLC system. The fabricated fibers were mechanically stable, and the extraction efficiency was almost unaffected. The in-tube SPME–HPLC analysis was accomplished in two steps: (i) introduction of a volume of 20 mL of diluted sample directly to the column through an injection valve; (ii) elution and separation of the analytes by flowing the mobile phase (0.1% formic acid/methanol 57/43 *v*/*v*) through the PEEK tube. Due of the high durability of the PEEK tube and extensive interspacing among the fibers, the elution can be performed using relatively high flow rates. High preconcentration factors (ca. 300-fold) were achieved, leading to LOD values as low as 0.05 ng mL^−1^. The drugs’ concentration was monitored in the rat plasma after oral administration of 1 mL of sulfonamide-containing tablet suspension of 0.1 mg mL^−1^. 

A biocompatible in-tube SPME based on poly(methacrylic acid-ethylene glycol dimethacrylate, MAA-EGDMA) monolithic capillary was published by Nie et al. for the analysis of angiotensin II receptor antagonists (candesartan, losartan, irbesartan, valsartan, telmisartan) in human plasma samples [62]. It was demonstrated that the enrichment capacity of the column was almost linearly increased by the extraction time. During the optimization of sample pH, telmisartan showed quite different extraction behavior compared to other “sartans”. This phenomenon was based on the mix-mode mechanism involved in the extraction procedure under different pH values. Sufficient elution of the analytes from the SPME column has been accomplished using a mixture of CH_3_COONa (5 mM, pH = 3.5)/CH_3_CN, 60/40 *v*/*v*. Excellent linearity was obtained for all analytes while the method is capable of determining the analytes in the range of 0.5–5000 ng mL^−1^ with satisfactory precision and accuracy. Furthermore, losartan has been extracted from plasma samples using a polypyrrole DES in-tube SPME [138]. The extraction sorbent was synthesized by electrochemical deposition on the inner walls of a stainless-steel capillary. The column exhibited adequate stability in relatively acidic and basic media, and it can be reused up to 450 times without decrease in extraction efficiency. The linearity of the method ranged between 0.1 and 500 μg L^−1^ and the inter- and intra-assay precisions (RSDs) varied in the range of 1.9–4.6%. An expanded work of the research of Yamini includes the determination of indomethacin in human plasma [95]. The analyte was selectively extracted from the biological matrixes using a nanostructured copolymer coating consisting of polypyrrole doped with ethylene glycol dimethacrylate. Relatively low LODs were achieved in the examined analyte in the range of 0.07–2.0 μg L^−1^. Stable and reproducible signals were obtained without being considerably influenced by interferences from endogenous compounds.

A group of potential neuroleptic drugs was determined using the commercial DB-17 capillary column on-line coupled with HPLC [46]. After optimization of the SPME parameters, the extraction efficiencies varied between 12.7% and 31.8% for moperone, spiroperidol, and pimozide and 1.08% and 4.86% for floropipamide, haloperidol, and bromperidol. For practical reasons, a cycle of 20 aspirating/dispensing processes was utilized to obtain the desired sensitivity in a reasonable analysis time. All analytes were found to be stable in plasma for at least 8 h at 4 °C and for 4 weeks at −80 °C. Freeze-thaw experiments indicated that the analytes were stable for up to three cycles.

An LC-MS/MS method in combination with in-tube SPME has been reported for the analysis of seven benzodiazepines (diazepam, nordiazepam, temazepam, oxazepam, 7-aminoflunitrazepam, *N*-desmethylflunitrazepam, and clonazepam) in serum samples [144]. A 60 cm long commercial GC capillary column (Supelco-Q plot) was utilized for the extraction of the analytes. The separation of benzodiazepines was carried out under ion-suppressed reversed-phase conditions by using 50 mM CH_3_COONH_4_/CH_3_OH, 40/60 *v*/*v* as mobile phase. Electrospray ionization in positive mode was utilized to produce the quasimolecular ions of the analytes and further MS/MS fragmentation. Depending on the structure of the compound, the characteristic ions appeared at different fragmentor voltages. According to the authors, the compounds containing hydroxyl groups fragmented at relatively lower fragmentor voltages. Low LODs were achieved, varying from 0.024 to 2 ng mL^−1^. Two years later, Mullet et al. developed a liquid chromatographic method for the analysis of the same drugs [85]. The bifunctionality of RAM (alkyl-diol-silica) columns permits the direct injection of serum samples without protein adsorption on the surface. At the same time, the analytes were trapped in the hydrophobic porous interior.

In order to enhance the selectivity of the determination of fluoxetine in serum specimens, an immunoaffinity in-tube SPME was exploited prior to LC/MS analysis [88]. The SPME tube was prepared by attaching an antibody in the fused silica capillary and used for the selective extraction of the analyte. The characterization of the antibody was made by Scatchard plot analysis. The immunosorbent phase was found to be stable after two weeks, when it was stored in phosphate buffered saline and 0.05% sodium azide solution. Adequate method sensitivity was achieved by using 20 “draw/inject” cycles. Although the salting out phenomenon increases the extraction efficiency, it causes blockage of the column by deposits in the tube [155]. 

Recently, a commercial capillary column has been utilized for in-tube SPME of caffeine and its metabolites in human serum, saliva, and urine [37]. A ZB-FFAP (100% nitroterephthalic-modified polyethylene glycol) commercial capillary column was utilized for the isolation of the analytes. One of the main advantages of the approach is the small sample amount required (only 2.5 μL of saliva, 6.25 μL of serum or 40 μL of urine), which is critical, especially in hospital laboratories, for preterm newborns. The analytical method was thoroughly validated according to FDA guidance [156]. The recoveries were reasonable for this type of analysis, ranging between 84% and 114%.

### 4.2. Urine

Urine is one of the most abundant biological matrices in terms of applicability, due to its availability and noninvasive collection compared to other biological fluids such as blood, serum, amniotic fluids, gastric contents. etc. It typically contains substantial amounts of dissolved inorganic salts and metabolites from various endogenous and exogenous compounds [153,157]. Although direct analysis of urine would be the best scenario, it is often not feasible due to inherent difficulties of this matrix, limiting the selectivity and the sensitivity of the analytical determinations. For instance, the high salt concentration of urine matrices leads to ionization suppression or enhancement during MS detection. Urine dilution is therefore the most commonly used sample treatment procedure prior to in-tube SPME. This step avoids the potential capillary clogging from the high salt concentrations of urine samples. In some cases, antioxidant agents (e.g., ascorbic acid or metabisulfite) may be added in samples in order to preserve the analyte [78,148].

Trace amounts of antipsychotic drugs (perphenazine, chlorpromazine) were determined in human urine samples by using an electrochemically controlled fiber-in-tube SPME [119]. The researchers utilized an almost identical instrumental configuration to that used in a previous approach [103], and they synthesized a nanostructured Cu-Cr-Al ternary layered double hydroxide/polythiophene coating by in-situ electrodeposition. The manufactured sorbent was characterized by X-ray diffraction (XRD), FTIR spectroscopy, and SEM. The total synthesis time was only 15 min. Satisfactory linearity was obtained in the range of 0.2–300 μg L^−1^, with a correlation coefficient greater than 0.9982. The sensitivity (expressed with LODs) of the method was adequate for this type of analysis. The authors worked on the same topic by developing an in-tube SPME-HPLC method for the determination of diclofenac and mefenamic acid in human urine samples [120]. A nanostructured polypyrrole-dodecyl benzene sulfonate (Ppy-DBS) material was coated on the inner surface of a stainless-steel tube by electrochemical deposition. A peristaltic pump was utilized to deliver the monomer solution from the inner surface of the stainless-steel tube. Compared to other SPME methodologies [158,159], the method provided lower LODs in a relatively short extraction time of 20 min. 

In 2018, Saito et al. published an LC-MS/MS analytical method for the determination of three urinary biomarkers (8-isoprostane, 8-hydroxy-2′-deoxyguanosine, and 3-nitro-l-tyrosine) in human urine [32]. These compounds are biomarkers of endogenous oxidative damage to lipids, DNA, and proteins. A Carboxen 1006 PLOT capillary column was employed for the sample pretreatment prior to analysis. Deprotonated ion [M – H]^−^ (ESI (-)ve mode) or protonated ion [M + H]^+^ (ESI (+)ve mode) and their prominent fragment ions were used as precursor and product ions for the quantitation of the analytes. Optimal separation was achieved using 5 mM HCOONH_4_/CH_3_OH 40/60, *v/v* at a flow rate of 0.1 mL min^−1^.

**Figure 6:**
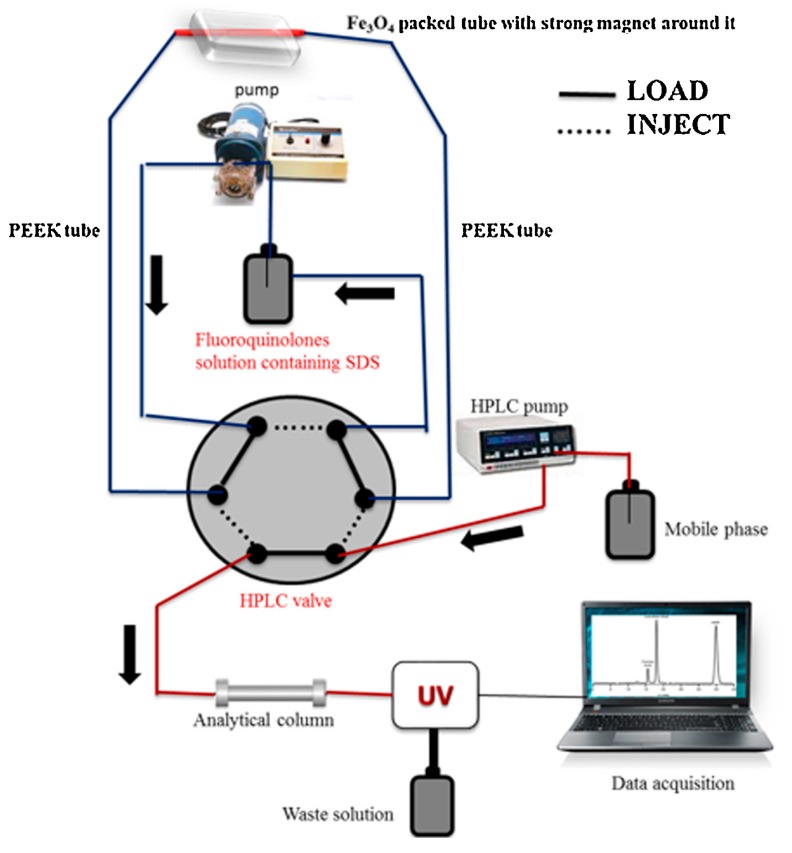
Instrumental setup for the determination of fluoroquinolones. Adopted from [147] with permissions.

Two analytical methods have been reported by the research group of Almadi for the determination of fluoroquinolones in human urine [146,147]. Magnetic nanoparticles functionalized with sodium dodecyl sulfate have been employed for in-tube SPME of moxifloxacin [146]. The authors concluded that the SDS molecules acted as a bridging agent between the analyte and the bare Fe_3_O_4_ material, and thus an enhancement of its retention was observed. The main advantages of the method included rapidity, simplicity, automation, and full sorbent collection after analysis. A more sophisticated work has been published by the same authors for the determination of ciprofloxacin, enrofloxacin, and ofloxacin using almost the same sorbent material [147]. All factors affecting the extraction performance were investigated and optimized using Plackett–Burman and Box–Behnken designs. The urine samples were firstly centrifuged and diluted 5-fold prior to SPME. Compared to other published microextraction techniques, the method was found to be advantageous in terms of limit of detection (0.01–0.05 μg L^−1^) and extraction time (13.5 min). A schematic representation of the in-tube-HPLC setup is depicted in Figure 6.

Catecholamines, such as dopamine and 5-hydroxytryptamine, play an important role in the nervous system for numerous organisms. A boronate affinity SPME coupled on-line to LC/MS/MS was reported for the determination of these compounds in urine samples [148]. The proposed material relies on covalent interactions and thus features with specific selectivity, eliminating the matrix effect. Faster extraction equilibrium was observed in this study due to the three reasons: (i) the boronic acid ligands are more accessible to analytes; (ii) lower flow rate of the sample solution employed; and (iii) thinner coatings favored fast equilibrium. The synthesized coatings were found to be able to endure for three months with at least 350 uses with an RSD between analyses less than 10%.

A sensitive LC-MS method has been proposed by Kataoka et al. for the analysis of nicotine, cotinine, and their related alkaloids in human urine and saliva [48]. After optimization of the method parameters, 25 draw/eject cycles with a sample size of 40 μL using a CP-Pora PLOT amine capillary column as the extraction device were adopted. The method was 20–46-fold more sensitive than the direct injection method (using 5 μL injection volume), due to the preconcentration step performed during the draw/eject cycles. The analytical method was used to evaluate the influence of the nicotine intake in active and passive smokers and after chewing Nicorette^®^ gum. The urinary cotinine content reached a maximum level after 4 h. Interestingly, it was found that urinary excretion of the analytes increased in nonsmokers associated with passive smoking. 

A group of anabolic steroids has been determined by using on-line in-tube SPME coupled with LC–MS [56]. The analytes were isolated from urine samples by exploiting a Supel-Q PLOT capillary column. After 20 draw/inject cycles with a sample volume of 40 μL, enrichment factors in the range of 20–33 were obtained. The analytes were separated on a Chromolith RP-18e column within 14 min using a mixture of 5 mM HCOONH_4_/CH_3_OH (35/65, *v*/*v*) at a flow rate of 1.0 mL min^−1^. The recoveries of these compounds were higher than 85% and the %RSD better than 8.3%. According to the authors, urinary excretion of glucuronide-conjugate of methyltestosterone is less than 0.1% of the dose, and it sufficiently reflects doping by use of methyltestosterone.

### 4.3. Saliva

Saliva is considered to be an “ultrafiltrate” of blood and a potential source of clinical information about patients. It contains various biomarkers that can provide critical information about oncological, cardiovascular, autoimmune, viral, and bacterial diseases. Saliva approximately contains 99% water, 0.3% protein (mainly enzymes), and 0.3% mucin (responsible for the stickiness of saliva) with the balance being salts [160]. Saliva sampling is a noninvasive procedure and it can be carried out by the patient. Details about the sampling of saliva, including devices and methods, has been recently reviewed by Bellagambi et al. [161].

An in-tube SPME application for saliva analysis has been described by Yasuhara et al. for the determination of stress-related steroid hormones [47]. Saliva samples were collected using Salisoft tubes, ultracentrifuged using a 30 kDa cut-off filter, and finally diluted prior to analysis. Optimum extraction performance was achieved by applying 25 draw/eject cycles of 40 μL of sample at a flow rate of 200 μL min^−1^ through a Supel-Q PLOT capillary column. Recoveries were quite satisfactory, ranging between 94.2% and 105%. This method is automated, simple, rapid, selective, and sensitive, and can be applied to the analyses of small volumes of saliva samples without pretreatment other than ultrafiltration. An interesting approach was reported by the research group of Kataoka, aiming at the quantitation of testosterones, cortisol, and dehydroepiandrosterone in saliva samples [55]. Compared to other coating materials, namely CP-Sil 5CB, CP-Sil 19CB, CP-Wax 52CB, and Carboxen 1010 PLOT, the Supel-Q PLOT coating had better extraction capabilities and thus was employed for the isolation of the analytes in the specific matrixes. The extracted compounds were simply desorbed from the capillary by passage of the mobile phase (dynamic desorption) consisting of 0.2% HCOOH/CH_3_CN 60/40, *v*/*v* at a flow rate of 0.2 mL min^−1^. The compounds were detected under MS/MS conditions, while an isotopic dilution approach was followed for the quantitation. The developed method has been utilized to study the changes in the concentration of the analytes in saliva in stress and fatigue load tests. According to their findings, cortisol levels increased 2.3–5.5-fold after mental stress and strength training, while dehydroepiandrosterone concentrations increased 1.67-fold after strength training. On the contrary, testosterone levels were almost unaffected. 

### 4.4. Miscellaneous

Breath analysis has attracted a considerable amount of scientific and clinical interest. It is a promising approach for the noninvasive assessment of inflammatory and oxidative stress biomarkers. Exhaled breath condensate (EBC) is a biological fluid collected by cooling exhaled air during tidal breathing [16,162]. 

Two different analytical methods have been proposed for the analysis of particular volatile aldehydes (butanal, pentanal, hexanal, heptanal, octanal, and nonanal) in EBC [102,151]. In 2015, Li et al. investigated the extraction capabilities of graphene/polyaniline electrodeposited coating for the isolation of the certain aldehydes [102]. The coating was attached on the internal surface of stainless-steel tube by a facile in situ electrodeposition. The characterization of the material was carried out by scanning electronic microscopy (SEM) and Fourier transform infrared spectroscopy (FTIR). A commercial device, the RTube^TM^, was utilized to collect the EBC samples. The analytes were derivatized with 2,4-dinitrophenylhydrazine prior to in-tube SPME. Enhanced method sensitivity and selectivity were obtained by measuring the derivatives at 360 nm. According to the authors, the average concentrations of butanal and heptanal in patients ranged from 25.2 to 99.3 nmol L^−1^ and 32.3 to 68.8 nmol L^−1^, respectively. However, there was a difference in pentanal and heptanal levels between healthy people and lung cancer patients. In the same fashion, Wang et al. took advantage of a polypyrrole/graphene (PPy/G) composite coating for the same type of analysis [151]. The merits of the method are comparable with those of [102] except for the LOD, which was ca. 100-fold higher. 

Hair is commonly utilized for drug analysis as its collection is noninvasive, and it provides a historical record of exposure, due to the relatively long lifetime of drugs in hair [160]. Sixteen heterocyclic amines were determined in hair by on-line in-tube SPME coupled to LC/MS/MS [36]. One of the goals of the approach was to provide data regarding the concentration levels of the studied biomarkers as an indicator of the exposure to cigarette smoke. Prior to SPME, the hair pieces were rinsed with CH_3_OH and then hydrolyzed at 100 °C in the presence of 1 M NaOH for 60 min followed by neutralization and dilution. The enrichment factors varied from 52 to 189 compared to the direct analysis of the compounds. The LOQs of the analytes were approximately 0.17–1.63 pg mg^−1^_._

## 5. Conclusions

Without any doubt, in-tube SPME is, in many ways, a state-of-the art sample preparation technique. It overcomes some disadvantages of traditional fiber-based SPME—sample consumption is minimal, it has a green character, high preconcentration factors can be achieved, it is readily interfaced with advanced analytical instrumentation, and it can be the basis of fully automated schemes. 

Apart from instrumental improvements, such as more effective coupling to separation techniques and mass spectrometry and flow-based automation, the heart of in-tube SPME research is materials and coatings. This is evident in the published reports from highly reputed research groups in this field. Novel materials improving the selectivity, preconcentration, and stability are mandatory to ensure the future of the technique. Some examples may include the stability of new materials at extreme pH values, coating with nanomaterials for improved performance, fabric-based absorbents, and last, but not least, the wider application of 3D printing technology. Of course, novel bioanalytical applications in new analytical problems should not be overlooked and underestimated since they play a critical role in the expansion of the technique and its adaptation in real-world applications. 

## Figures and Tables

**Table 1:** Bioanalytical HPLC applications of in-tube SPME for the determination of drugs.

Analyte	Sample	SPME Material	SPME Mode	Detection	LOD/LOQ	Year	Ref
Cannabidiol, Δ9-tetrahydrocannabinol	Human plasma	Dummy molecularly imprinted monolithic capillary	In-valve	MS/MS	NM ^1^/10 ng mL^−1^	2020	[134]
Chlopromazine, clozapine, quetiapine, olanzapine, and their metabolites	Human plasma	Butyl methacrylate-co-ethylene glycol dimethacrylate monolith	In-valve	MS/MS	NM/10 ng mL^−1^	2019	[135]
Anandamide, 2-arachidonoyl glycerol	Human plasma	Polymeric ionic liquid open tubular capillary column	In-valve	MS/MS	NM/0.05, 0.10 ng mL^−1^	2019	[136]
Amino acids, neurotransmitters	Human plasma	Dual ligand sol–gel organic-silica hybrid monolithic capillary	In-valve	MS/MS	NM/6–360 nmol mL^−1^	2019	[137]
Ketoprofen, flurbiprofen, diclofenac	Human plasma	Poly(deep eutectic solvent) monolithic column	In-valve	UV	0.05–0.5/0.2–2 ng mL^−1^	2018	[79]
Losartan	Human plasma, urine	Polypyrrole-deep eutectic solvent coated capillary	In-valve	UV	0.2, 0.5 μg L^−1^/NM	2018	[138]
Jatorrhizine, palmatine, berberine	Rat plasma	immobilized Graphene oxide on PEEK tube	In-valve	MS/MS	0.1–0.3 pg mL^−1^	2017	[139]
Amitriptyline, imipramine, chlorpromazine	Human plasma	indole-thiophene copolymer nanocomposite	In-valve	UV	40 ng mL^−1^/80 ng mL^−1^	2017	[140]
Amitriptyline, doxepin	Human plasma, urine	Polythiophene/graphene oxide (PTh/GO) nanostructured coating	In-valve	UV	0.3, 0.5/2.3, 2.9 ng mL^−1^	2016	[103]
Sulfadiazine, sulfadimidine, sulfamethoxazole	Rat plasma	Poly(3,4-ethylenedioxythiophene)	In-valve	UV	0.002–0.05/0.01–0.25 ng mL^−1^	2016	[141]
Berberine, palmatine, jatrorrhizine	Rat plasma	Poly(acrylamide–ethylene glycol dimethacrylate)monolith	In-valve	UV	0.01/0.03 ng mL^−1^	2013	[142]
Glycoproteins	Rat plasma	Poly(vinylphenylboronic acid–ethylene glycol dimethacrylate) monolithic material	In-valve	UV	0.01 μg mL^−1^/NM	2018	[82]
Interferon alpha 2a	Human plasma	Molecularly imprinted polymer	Draw-inject	FLD	NM/8 ng mL^−1^	2013	[94]
Interferon alpha 2a	Human plasma	Monoclonal anti-interferon 2a antibody	Draw-inject	FLD	NM/0.006 MIU mL^−1^	2013	[87]
Ketoprofen, fenbufen, ibuprofen	Human plasma	Poly(4-vinylpyridine-co-ethylene dimethacrylate) monolith	In-valve	UV	2.01–4.77/6.70–15.9 ng mL^−1^	2012	[80]
Lidocaine and its metabolite	Human plasma	14% cyanopropylphenyl methylpolysiloxane	Draw-inject	UV	15, 20/50 ng mL^−1^	2012	[33]
Rifampicin	Human plasma	Polyethylene glycol	Draw-inject	UV	MN/0.1 μg mL^−1^	2011	[34]
Interferon alpha 2a	Human plasma	Restricted access material (protein-coated silica)	Draw-inject	FLD	NM/0.06 MIU mL^−1^	2011	[86]
Antidepressants	Human plasma, urine	Hybrid organic–inorganic silica monolith with cyanoethyl functional groups	In-valve	MS	0.06–2.84/0.19–9.45 ng mL^−1^	2010	[83]
Fluoxetine, norfluoxetine	Human plasma	Polypyrrole-coated capillary	Draw-inject	RF	NM/10–15 ng mL^−1^	2009	[125]
Moperone, floropipamide, haloperidol, spiroperidol, bromperidol, pimozide	Human plasma	DB-17	Draw-inject	MS/MS	0.03–0.2/0.1–0.5 ng mL^−1^	2009	[46]
Mirtazapine, citalopram, paroxetine, duloxetine, fluoxetine, sertraline	Human plasma	OV-1701	Draw-inject	UV	5–20/20–50 ng mL^−1^	2008	[35]
Candesartan, losartan, irbesartan, valsartan, telmisartan	Human plasma, urine	Poly(MAA-EGDMA) monolithic capillary	In-valve	FLD	0.1–15.3/0.4–51 ng mL^−1^	2005	[62]
Camptothecin, 10-hydroxycamptothecin	Human plasma	Poly(MAA-EGDMA) monolithic capillary column	In-valve	UV	1.79–2.62/5.96–8.73 ng mL^−1^	2005	[66]
Verapamil metabolites	Human plasma, urine	Polypyrrole-coated capillary	Draw-inject	UV, MS	52–83 ng mL^−1^ (UV), 5–8 ng mL^−1^ (MS)/NM	2002	[121]
Indomethacin	Human plasma, urine, blood	Nanostructured copolymer coating consisting of polypyrrole doped with ethylene glycol dimethacrylate	In-valve	UV	0.6–2.0 μg L^−1^/NM	2016	[95]
Theobromine, theophylline, caffeine	Human serum	Poly(methacrylic acid–ethylene glycol dimethacrylate) monolithic	In-valve	UV	6.5–12.0/21.5–39.6 ng mL^−1^	2004	[60]
Oxazepam, temazepam, nordazepam, diazepam	Human serum	Restricted access material (RAM), alkyl-diol-silica (ADS),	Draw-inject	UV	22–29/74–98 ng mL^−1^	2002	[85]
Fluoxetine	Human serum	Immunoaffinity-based (BSA-fluoxetine conjugate)	Draw-inject	MS	NM/5 ng mL^−1^	2007	[88]
Glycoprotein	Human serum	Boronate-functionalized molecularly imprinted monolithic column	In-valve	UV	NM	2013	[143]
Theobromine, paraxanthine, theophylline, caffeine	Human serum	ZB-FFAP (100% nitroterephthalic modified polyethylene glycol).	In-valve	UV	0.1–0.5/0.4–1.5 μg mL^−1^	2020	[37]
Benzodiazepines	Human serum, urine	Supelco-Q plot capillary column	Draw-inject	MS	0.02–2/0.5–2 ng mL^−1^	2000	[144]
Beta-blockers	Human serum, urine	Omegawax 250 capillary	Draw-inject	MS	0.1–1.2 ng mL^−1^/NM	1999	[40]
Stimulants, beta-blockers	Human serum, urine	Omegawax 250 capillary	Draw-inject	MS	0.1–1.2 ng mL^−1^/NM	2000	[145]
Propranolol	Human serum	Molecularly imprinted polymer	Draw-inject	UV	0.32 μg mL^−1^	2001	[93]
17β-Estradiol, estrone, ethinyl estradiol, progesterone, estriol	Human urine	NH_2_-MIL-53(Al)-polymer monolithic column	In-valve	UV-FLD	0.002–0.04 μg L^−1^/NM	2017	[78]
Naproxen	Human urine	Polypyrrole (PPy)-coated	In-valve	UV	0.07 μg L^−1^	2015	[122]
Moxifloxacin	Human urine	Fe_3_O_4_ nanoparticles-packed	In-valve	UV	0.03 μg L^−1^/NM	2015	[146]
Ciprofloxacin, enrofloxacin, ofloxacin	Human urine	Sodium dodecyl sulfate coated Fe_3_O_4_ nanoparticles	In-valve	UV	0.01–0.05 μg L^−1^	2015	[147]
Dopamine, 5-hydroxytryptamine	Human urine	Boronate affinity solid phase microextraction	In-valve	MS/MS	1.2/4.0 ng mL^−1^	2010	[148]
Nicotine, cotinine, nornicotine, anabasine, anatabine	Human urine, saliva	CP-Pora PLOT amine capillary column	Draw-inject	MS	0.015–0.040 ng mL^−1^/NM	2009	[48]
Ketoprofen, fenbufen, ibuprofen	Human urine	Beta-cyclodextrin coated capillary column	Draw-inject	UV	18–38 ng mL^−1^/NM	2005	[117]
Ketamine	Human urine	Poly(methacrylic acid- ethylene glycol dimethacrylate) monolithic capillary	In-valve	UV	6.4 ng mL^−1^/NM	2004	[68]
Stimulants	Human urine, hair	Polypyrrole coated capillary column	Draw-inject	MS	8–56 ng L^−1^/NM	2001	[124]
Diclofenac, mefenamic acid	Human urine, plasma	Nanostructured polypyrrole	In-valve	UV	0.08–1.6 μg L^−1^/NM	2018	[120]
Urinary biomarkers (8-isoprostane, 8-hydroxy-2′-deoxyguanosine, 3-nitro-L-tyrosine)	Human urine	Carboxen 1006 PLOT capillary column	Draw-inject	MS/MS	3.4–21.5 pg mL^−1^/0.02 ng mL^−1^	2018	[32]
Heterocyclic amines	Human urine	Supel-Q PLOT capillary column	Draw-inject	MS/MS	NM/1.7–4.1 pg mL^−1^	2014	[54]
8-hydroxy-2′-deoxyguanosine, 3-hydroxyphenanthrene, 1-hydroxypyrene	Human urine	Graphene oxide, poly(3,4-ethylenedioxythiophene), poly- pyrrole	In-valve	MS	0.004–0.041/0.016–0.135 ng mL^−1^	2019	[149]
8-hydroxy-2′-deoxyguanosine	Human urine	Carboxen 1006 PLOT capillary column	Draw-inject	MS/MS	8.3 pg mL^−1^/NM	2016	[49]
Perphenazine, chlorpromazine	Human urine, plasma	nanostructured Cu-Cr-Al ternary layered double hydroxide/polythiophene coating	In-valve	UV	0.2–0.8 μg L^−1^/NM	2018	[119]
Nornicotine, anatabine, anabasine, nicotine, cotinine	Human urine, saliva	CP-Pora PLOT amine capillary column	Draw-inject	MS	115–40 pg mL^−1^/NM	2009	[48]
Cortisol, dehydroepiandrosterone	Saliva	Supel-Q PLOT capillary column	Draw-inject	MS/MS	0.9–12 pg mL^−1^/NM	2012	[47]
Testosterone, cortisol, dehydroepiandrosterone	Saliva	Supel-Q PLOT capillary column	Draw-inject	MS/MS	NM/0.01–0.29 ng mL^−1^	2013	[55]
Oxytocin	Saliva	Supel-Q PLOT capillary column	Draw-inject	MS/MS	4 pg mL^−1^/NM	2015	[150]
Butanal, pentanal, hexanal, heptanal, octanal and nonanal	Exhaled breath condensate	Graphene/polyaniline (G/PANI) electrodeposited coating	In-valve	UV	0.02–0.04/0.07–0.13 nmol L^−1^	2015	[102]
Butanal, pentanal, hexanal, heptanal, octanal and nonanal	Exhaled breath condensate	Polypyrrole/graphene (PPy/G) composite coating	In-valve	UV	2.3–3.3/7.7–12.3 nmol L^−1^	2015	[151]
Polycyclic aromatic hydrocarbons	Hair	CP-Sil 19CB (14% cyanopropyl phenyl methylsilicone)	In-valve	FLD	0.5–20.4 pg mL^−1^/NM	2015	[152]
Heterocyclic amines	Hair	Supel-Q PLOT capillary column	Draw-inject	MS/MS	0.10–0.78 pg mL^−1^/NM	2013	[36]

^1^ NM: not mentioned
